# Epidemiologic and economic burden of HPV diseases in Spain: implication of additional 5 types from the 9-valent vaccine

**DOI:** 10.1186/s13027-018-0187-4

**Published:** 2018-05-02

**Authors:** Noelia López, Aureli Torné, Agustín Franco, María San-Martin, Elisabet Viayna, Carmen Barrull, Nuria Perulero

**Affiliations:** 1 Medical Affairs, MSD, Madrid, Spain; 2 Gynecologic Oncology Unit, Institut Clinic of Gynecology, Obstetrics and Neonatology (ICGON), Hospital Clínic-Institut d′Investigacions Biomèdiques August Pi i Sunyer (IDIBAPS), Faculty of Medicine, University of Barcelona, Barcelona, Spain; 3 Diagnostic Unit of Urology, Clinic Institute of Nephrology and Urology (ICNU) Hospital Clínic Barcelona, University of Barcelona, Barcelona, Spain; 4 Health Economics and Outcomes Research, Real World Insights, IQVIA, Barcelona, Spain

**Keywords:** Burden of disease, Human papillomavirus, Vaccine, Precancerous lesions, Cancer

## Abstract

**Background:**

A new nonavalent human papillomavirus (HPV) vaccine that includes genotypes 6/11/16/18/31/33/45/52/58 has been recently approved in Spain. A previous study has shown that attributable fraction of HPV related diseases in Spain is consistent with that reported in European and global studies. The aim of the present study was to estimate the annual direct costs associated to the following HPV-related diseases: genital warts, high grade precancerous lesions and cancer of cervix, vulva, vagina, anus and penis and head and neck cancer, caused by genotypes included in the nonavalent (9vHPV) and quadrivalent vaccines (4vHPV), in Spanish men and women.

**Methods:**

Cancer registries and epidemiological studies were used to estimate the number of new annual cases of genital warts, anogenital precancerous lesions and cancer of cervix, vulva, vagina, anus, penis and head and neck, as well as the fraction attributable to HPV infection and to genotypes targeted by both vaccines in Spain. Costs per patient for each disease were obtained from the literature. In addition, 142 specialists were surveyed to estimate cost per patient of vulvar, vaginal, anal and penile precancerous lesions. The annual burden of diseases attributable to types targeted by both vaccines was estimated and compared. All results were validated by a panel of experts.

**Results:**

In 2016, new genital warts, precancerous lesions and cancers attributable to types targeted by the 9vHPV were estimated at 49,251, 29,405 and 3381, respectively. Among them, 12,597 new precancerous lesions and 530 new cancers were related to the 5 additional types covered by the 9vHPV. Annual cost of new cases of these diseases associated to types targeted by the 4vHPV and 9vHPV were estimated at 116.7 and 150.9 million € for the Spanish National Health Service (NHS), respectively (2017 €).

**Conclusions:**

HPV-related diseases represent a major burden for the Spanish NHS. Annual new cases and costs related to the 5 additional types from the 9vHPV represent a significant burden compared with that associated to types included in the 4vHPV.

**Electronic supplementary material:**

The online version of this article (10.1186/s13027-018-0187-4) contains supplementary material, which is available to authorized users.

## Background

The fact that human papillomavirus (HPV) was recognized as a necessary cause of cervical cancer led to the development of a first generation of HPV vaccines: a bivalent and a 4-valent (4vHPV), capable of preventing diseases associated to genotypes 16/18 and 6/11/16/18, respectively. In 2007, Spanish national vaccination program included HPV-immunization for all girls aged 14 years old [1]. Although vaccination programs initially intended to prevent cervical cancer in women, recent studies have proved implication of HPV in many other diseases which may affect both men and women, namely: vulvar, vaginal, anal and penile precancerous lesions and cancer as well as some head and neck cancers, and other benign diseases such as genital warts. The evidence of HPV implications in non-cervical related diseases, has led to more ambitious vaccination strategies with the aim of controlling HPV infection [2, 3].

A HPV vaccine 9-valent (9vHPV) that includes the following genotypes: 6/11/16/18/31/33/45/52/58 has been approved by the European Medicines Agency and the Spanish Agency of Medicine and Medical Products (AEMPS). This vaccine has proved to prevent 97% of precancerous lesions of cervix, vulva and vagina caused by its 5 additional genotypes (31/33/45/52/58), while maintaining protection against the 4 genotypes targeted by the former 4vHPV [4].

Attributable fraction of HPV related diseases to these new 5 types has been already reported at global and European level [3, 5]. In Spain, results from a literature review which aimed to identify evidence of the epidemiological burden of HPV-related diseases and the fraction of each lesion attributable to the specific genotypes targeted by the 4vHPV and 9vHPV has been recently published [6]. This study showed that attributable fraction of HPV related diseases in Spain is consistent with that reported in previous European and global studies [2, 7–10].

The aim of the present study was to estimate the annual direct costs associated to the following HPV related diseases: genital warts, high grade precancerous lesions and cancer of cervix, vulva, vagina, anus and penis and head and neck cancer, caused by genotypes included in the 9vHPV and 4vHPV, in Spanish men and women.

## Methods

### Estimation of the annual number of new cases of genital warts, precancerous lesions and cancers in Spain

Incidence rate for genital warts was obtained from an epidemiological study published by Castellsague et al. in 2009 [11].

In order to estimate incidence rates for each type of cancer, the Cancer Spanish Registry REDECAN was searched [12]. REDECAN meets the International Agency for Research in Cancer quality standard and is used to estimate the incidence of major cancers in Spain in the Cancer Incidence in Five Continents database [13]. Incidence rate for cervical cancer (International Classification of Diseases 10th revision [ICD-10] code: C53) was already estimated for the whole Spanish territory. For all other cancer sites (vulva [ICD-10: C51], vagina [ICD-10: C52], anus [ICD-10: C21], penis [ICD-10: C60], oral cavity [tongue, other and unspecified parts ICD-10: C02; mouth ICD-10: C03–06], nasopharynx [ICD-10: C11]; oropharynx [base of tongue ICD-10: C01; tonsil ICD-10: C09; oropharynx ICD-10: C10]; hypopharynx [ICD-10: C12–13]; pharynx [ICD-10: C14]; larynx [ICD-10:C32]), for which only data from each regional registry was available, a weighted average of incidence rates estimated by each registry was calculated, considering their geographical coverage at the time data was collected and then extrapolated to the whole Spanish territory [12, 13].

Literature reporting incidence rates for precancerous lesions other than cervix in Spanish population was not available. Only one study estimating the number of newly diagnosed cervical precancerous lesions in Spain was identified [14]. In the absence of specific data for Spanish population, the ratio between incidence rates for high grade precancerous lesions of cervix (CIN2/3), vulva (VIN2/3), vagina (VaIN2/3), anus (AIN2/3) and penis (PIN2/3) and cancer incidence rate for the corresponding locations was calculated for those countries for which robust population-based studies had been conducted [15–17] and applied to the Spanish cancer incidence rates to estimate incidence rates for precancerous lesions.

The annual number of each type of lesion, together with the 95% confidence intervals were estimated using the latest definitive Spanish population reported by the Spanish Statistical Office (December 2016) (men: 22,813,635, women: 23,654,467, total: 46,468,102) [18].

### Estimation of the number of HPV-related lesions attributable to genotypes targeted by the 4vHPV and 9vHPV

Except for cervical cancer, for which a study describing HPV-genotype distribution in cervical cancer cases in Spain published in 2012 was available (Table 1) [21], the fraction of each disease attributable to HPV infection and to genotypes targeted by the 4vHPV and 9vHPV were extracted from robust international studies. Specific fraction attributable to HPV for the European region was extracted in each case [2, 7–10, 22, 23]. All these studies use three different markers of viral presence and activity (viral DNA, mRNA, and/or overexpression of p16) to estimate the HPV attributable fraction. However, except for invasive vulvar cancer [8], detail on the percentage of cases that were positive for HPV DNA and either mRNA and/or p16 overexpression, thus proving the prevalence of oncogenically active HPV infection for each genotype, was not directly available from these publications [2, 7, 9, 10, 21–23]. In this context, in order to avoid an overestimation, except for vulvar cancer for which attributable fraction was estimated based on HPV DNA and p16 positivity, for all other cancer sites, genotypes 6 and 11 were excluded from the estimation of the fraction of each cancer attributable to the genotypes targeted by each vaccine. Also, in order to avoid overestimating the contribution of each HPV type due to multiple infections, contribution of individual HPV types to multiple infection cases was calculated under a weighting attribution which was proportional to the prevalence of each particular type in single-infection [19, 20]. This is further described in the original publications from which the fraction attributable to each genotype has been extracted [2, 7, 9, 10, 21–23].

**Table 1:** HPV prevalence, fraction attributable to genotypes targeted by the 4-valent and nonavalent vaccines and potential absolute and relative benefit of the 9-valent vaccine compared to the 4-valent for each type of lesion

Lesion [sources]	HPV prevalence	HPV 6/11/16/18 attributable fraction among HPV+ cases	HPV 6/11/16/18/31/33/45/52/58 attributable fraction among HPV+ cases	HPV 31/33/45/52/58 absolute (relative) attributable fraction among HPV+ cases
Genital warts [2]	100%	90%	90%	0% (0%)
CIN 2/3 [2]	100%	45.5%	82.3%	36.8% (80.9%)
VIN2/3 [8]	86.9%	80.9%	93.9%	13.0% (16.1%)
VaIN 2/3 [9]	98.0%	65.7%	79.0%	13.3% (20.2%)
AIN 2/3 [22]	95.7%	80.5%	87.8%	7.3% (9.1%)
PIN 2/3 [10]	89.1%	81.0%	91.9%	10.9% (13.4%)
Cervical cancer [21]	100%	72.4%(a)	88.3%(a)	15.9% (21.9%)(a)
Vulvar cancer [8]	18.3% (b)	78.0%	91.6%	13.6% (17.4%)
Vaginal cancer [9]	71.0%	64.0%(a)	84.2%(a)	20.2% (31.6%)(a)
Anal cancer [22]	87.6%	84.3%(a)	92.3%(a)	8.0% (9.5%)(a)
Penile cancer [10]	32.2%	70.2%(a)	79.4%(a)	9.2% (13.1%)(a)
Oral cavity [23]	7.4%	72.0%(a)	80.6%(a)	8.6% (11.9%)(a)
Nasopharynx [23]	7,9%	75.0%(a)	87.5%(a)	12.5% (16.7%)(a)
Oropharynx cancer [23]	24.9%	85.2%(a)	89.7%(a)	4.5% (5.3%)(a)
Hypopharynx cancer [23]	3.9%	80.0%(a)	100%(a)	20.0% (25.0%)(a)
Pharynx cancer [23]	21.4%	66.7%(a)	66.7%(a)	0% (0%)(a)
Larynx cancer [23]	5.7%	57.6%(a)	74.6%(a)	17.0% (29.5%)(a)

AIN: anal intraepithelial neoplasia; CIN: cervical intraepithelial neoplasia; HPV: human papillomavirus; PIN: penile intraepithelial neoplasia; VaIN: vaginal intraepithelial neoplasia; VIN: vulvar intraepithelial neoplasia

(a) Genotypes 6 and 11 not included; (b) HPV attributable fraction estimated based on HPV DNA and p16 positivity

### Estimation of direct costs of managing HPV-related diseases in Spain

In November 2015, a literature review in different databases such as Medline, Embase and Cochrane was conducted through the OVID platform in order to identify studies that estimated the incidence and prevalence of HPV genotypes in Spanish population, as well as the direct costs associated to the management of the following diseases: genital warts, cancer and precancerous lesions of cervix, vulva, vagina, anus, penis and head and neck published between January 1st 1995 and November 30th 2015. Methodology and results related to incidence and prevalence of HPV in Spanish population have been already published [6]. Results related to direct costs associated to the management of aforementioned disease have not been previously disclosed and will be reported herein. For this purpose, the search strategy was again executed on the 25th of June of 2017 in order to ensure no additional studies had been published. A specific search strategy was designed combining search terms related to the predefined diseases of interest which were combined with economic search terms. The filter designed by Valderas et al. [24] was used in order to identify studies including Spanish population. The search strategy is detailed as Additional file 1: Table S1.

References reporting direct costs for Spanish NHS for the aforementioned diseases were included and costs were transformed to € of 2017 according to Spanish Consumer Price Index (CPI) [18]. For those diseases for which more than one study reported annual direct cost for its management, the most robust study was selected. In this context, original full articles (rather than literature review or conference abstracts), including larger sample of patients, considering a higher number of resources, with a longer follow up period or with a higher representativeness of the Spanish territory (on the basis of the number of Autonomous Communities included) were prioritized.

For those diseases for which no direct costs were identified through the literature review (VIN2/3, VaIN2/3, AIN 2/3 and PIN2/3), a survey on use of resources for these type of lesions was formulated for which respondents were gynecologists, coloproctologists and urologists from all around Spain with expertise in the management of the aforementioned diseases. The survey was designed to retrieve annual use of resources in terms of general practitioner, specialists and emergency visits, days of hospitalization, diagnosis tests performed (cytologies, HPV detection test, biopsies) and treatment (surgical interventions, laser procedures and pharmacological treatment). The survey was validated by a panel of experts including one gynecologist, one urologist, one coloproctologist and one oncologist who tested its feasibility. The survey consisted of 2 main sections. The first section included two screening questions to identify the medical specialty of the participant and the number of patients with VIN2/3, VaIN2/3, AIN2/3 and/or PIN2/3 diagnosed or treated during the last 18 months. Only those gynecologists who had treated or diagnosed a minimum of 3 VIN2/3 and/or 5 VaIN2/3, coloproctologists who had treated or diagnosed a minimum of 3 AIN2/3 and urologists who had treated or diagnosed at least 5 PIN2/3 during the previous 18 months were allowed to participate. The second section included questions about resources used for the diagnosis and management of patients with the aforementioned pathologies. For each type of lesion, only aggregated data was collected, no individual use of resources was retrieved. Questions included number of visits, length of hospital stay, emergency visits, diagnoses test and treatments (surgical, laser or pharmacological treatments). No indirect costs were included. The survey was sent by email to 180 gynecologists, 380 coloproctologists and 201 urologists from the Mebos IQVIA panel. This panel includes healthcare professionals from all medical specialties and all Spanish regions, who work in public and/or private setting. A copy of the survey is presented as Additional file 1: Table S2.

In order to transform the use of resources into cost per patient for each type of lesion, the cost of each intervention, visit and procedure was searched in a Spanish costs database [25]. All costs were transformed to 2017 € [18]. Costs of pharmacological treatments were extracted from the Official College of Pharmacists [26].

Annual direct costs for each disease estimated through the surveys were validated by a multidisciplinary panel of experts.

### Estimation of the epidemiological and economic burden associated to the 9 and 4 types targeted by the 9vHPV and 4vHPV

In order to estimate the burden of HPV-related diseases associated to types targeted by each vaccine, the number of new annual cases of each disease was multiplied by the fraction attributable to HPV infection and by the fraction attributable to the specific genotypes extracted from the aforementioned studies [2, 7–10, 22, 23]. Those cases associated to types targeted by each vaccine were multiplied by the annual cost per patient estimated for each disease and compared.

## Results

### Estimation of the number of HPV-related lesions attributable to the genotypes targeted by the 4vHPV and 9vHPV

Annual incidence rates and estimated number of new annual cases in 2016 in Spain for each disease are presented in Table 2. Genital warts were the most common disease (54,723), followed by cervical precancerous lesions (33,594) and cervical cancer (2389) in women and larynx (3815) and oral cavity (1723) cancers in men.

**Table 2:** Crude incidence rates and estimated number of new annual cases of genital warts, precancerous lesions and cancer in men and women in Spain

Lesion [sources]	ICD-10	Crude annual incidence rate per 100.000 men or women	Estimated number of new annual cases in 2016 in Spain (95% CI)
Genital warts (men) [11]	NA	136.60	31,163 (30,806–31,497)
Genital warts (women) [11]	NA	99.60	23,560 (23,238–23,839)
CIN 2/3 [13,15]	NA	142.02	33,594 (33,235–33,953)
VIN2/3 [13,15]	NA	6.02	1424 (1350–1498)
VaIN 2/3 [13,15]	NA	1.02	241 (210–271)
AIN 2/3 (men) [13, 16]	NA	0.57	130 (108–153)
AIN 2/3 (women) [13, 16]	NA	0.27	63 (47–78)
PIN 2/3 [13, 17]	NA	1.32	302 (268–336)
Cervical cancer [13]	C53	10.10	2389 (2293–2485)
Vulvar cancer [12, 13]	C51	2.77	655 (556–754)
Vaginal cancer [12, 13]	C52	0.47	111 (70–151)
Anal cancer (men) [12, 13]	C21	0.86	197 (143–250)
Anal cancer (women) [12, 13]	C21	0.68	160 (111–209)
Penile cancer [12, 13]	C60	1.91	436 (356–516)
Oral cavity cancer (men) [12, 13]	C02–06	7.55	1724 (1492–1955)
Oral cavity cancer (women) [12, 13]	C02–06	0.64	736 (574–876)
Nasopharynx cancer (men) [12, 13]	C11	1.54	352 (277–426)
Nasopharynx cancer (women) [12, 13]	C11	0.45	106 (65–148)
Oropharynx cancer (men) [12, 13]	C01, C09, C10	1.64	1066 (841–1290)
Oropharynx cancer (women) [12, 13]	C01, C09, C10	0.10	152 (68–233)
Hypopharynx cancer (men) [12, 13]	C12–13	3.62	826 (711–940)
Hypopharynx cancer (women) [12, 13]	C12–13	0.25	59 (28–89)
Pharynx cancer (men) [12, 13]	C14	1.05	240 (179–302)
Pharynx cancer (women) [12, 13]	C14	0.13	32 (9–54)
Larynx cancer (men) [12, 13]	C32	16.72	3815 (3569–4060)
Larynx cancer (women) [12, 13]	C32	0.92	217 (158–276)

95% CI: 95% confidence interval; NA: not applicable; AIN: anal intraepithelial neoplasia; CIN: cervical intraepithelial neoplasia; HPV: human papillomavirus; ICD: International Classification of Diseases; PIN: penile intraepithelial neoplasia; VaIN: vaginal intraepithelial neoplasia; VIN: vulvar intraepithelial neoplasia

New cases of genital warts, precancerous lesions and cancers attributable to types targeted by the 9vHPV was estimated at 49,251, 29,405 and 3381, respectively, in 2016 in Spain. Out of which, all 49,251 new genital warts were attributable to the types targeted by the 4vHPV, whereas for precancerous lesions and cancer 12,597 (42,8%) and 530 (15,7%) new cases were attributable to the 5 additional types from the 9vHPV, respectively.

### Estimation of direct costs of managing HPV-related lesions in Spain

Studies identified through the literature review, resources considered and total direct costs for the Spanish NHS (2017 €) are reported as Additional file 1: Table S3. The most robust studies are summarized in Table 3 [11, 14, 27–30].

**Table 3:** Direct costs of the management of each type on lesion in Spain reported in the literature and use of resources considered

Lesion [sources]	Cost (2017 €)	Use of resources
Genital warts [11]	1036€	Visits with specialists, diagnosis tests, pharmacological and other treatments and hospitalizations.
CIN 2/3 [14]	CIN2:2022€	Visits with gynecologists, diagnosis tests, pharmacological, laser and surgical treatments, and medical complications.
	CIN 3: 2600€	
Cervical cancer [27]	8760€	Diagnosis (colposcopy, cytology, HPV DNA testing), treatment (LEEP/LLETZ, hysterectomy, conization, laser destruction, radiotherapy and chemotherapy)
Vulvar cancer [28]	12,994€	Direct cost of diagnosis, treatment and hospitalization
Vaginal cancer [28]	10,664€	
Anal cancer (men) [29]	7790€	Direct costs of hospitalized patients based on Diagnosis-related groups
Anal cancer (women) [29]	7481€	
Penile cancer [29]	7135€	
Oral cavity cancer (men) [30]	7984€	
Oral cavity cancer (women) [30]	8198€	
Nasopharynx cancer (men) [30]	8086€	
Nasopharynx cancer (women) [30]	6708€	
Oropharynx cancer (other) (men) [30]	7373€	
Oropharynx cancer (other) (women) [30]	7462€	
Hypopharynx cancer (men) [30]	8213€	
Hypopharynx cancer (women) [30]	7838€	
Pharynx cancer (men) [30]	7478€	
Pharynx cancer (women) [30]	8618€	
Larynx cancer (men) [30]	8652€	
Larynx cancer (women) [30]	9257€	

CIN: cervical intraepithelial neoplasia; DNA: deoxyribonucleic acid; HPV: human papillomavirus; LEEP: loop electrosurgical excision procedure; LLETZ: large loop excision on the transformation zone

Regarding those costs associated to VIN2/3, VaIN2/3, AIN2/3 and PIN2/3 estimated through surveys to specialists, 33 and 44 gynecologists provided data on the management of 99 VIN and 230 VaIN patients, respectively, 24 coloproctologists provided data from 72 patients with AIN2/3 and 56 urologists reported data on the management of 280 patients with PIN2/3. Only aggregated data was collected.

**Fig. 1:**
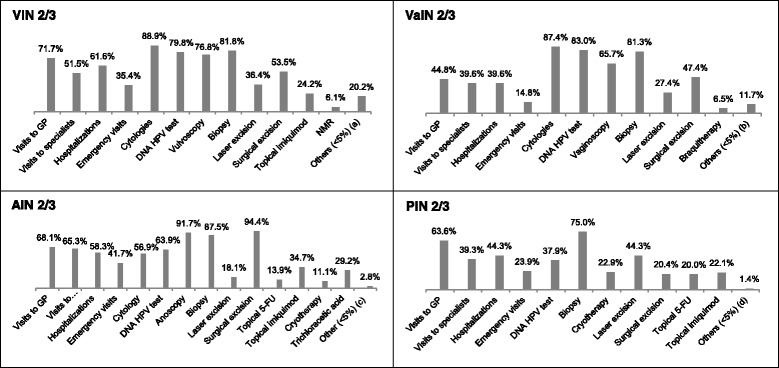
Percentage of patients who used each resource at least once for each type of lesion, according to the information provided by surveyed specialists. (a) general analytics (3,0%); anoscopy (3,0%), cervical culture (3,0%), colposcopy (3,0%), sexually transmitted disease serology (3,0%), vaginoscopy (3,0%), trichloracetic acid (1,0%), cryotherapy (1,0%). (b) vulvoscopy (2,2%), NMR (1,7%), colposcopy (1,7%), conization (1,7%), cervical culture (1,3%), cryotherapy (1,3%), 5-FU (0,9%), imiquimod (0,4%), trichloracetic acid (0,4%). (c) IR (2.8%). (d) NMR (1.4%). 5-FU: 5-fluorouracil; AIN: anal intraepithelial neoplasia; DNA: deoxyribonucleic acid; GP: general practitioner; HVP: human papillomavirus; IR: infrared; NMR: nuclear magnetic resonance; PIN: penile intraepithelial neoplasia VaIN: vaginal intraepithelial neoplasia; VIN: vulvar intraepithelial neoplasia

Figure 1 shows the percentage of patients with each type of lesion who used each resource at least once according to the surveyed specialists. 61.6%, 39.6%, 58.3% and 44.3% of patients with VIN2/3, VaIN2/3, AIN2/3 and PIN2/3, respectively, required hospitalizations. Cytologies were the most common test performed in patients with VIN2/3 (88.9%) and VaIN2/3 (87.4%), whereas biopsies were the most common within patients with AIN2/3 (91.7%) and PIN2/3 (75.0%). DNA HPV test was conducted in 79.8%, 83.0%, 63.9% and 37.9% of patients with VIN2/3, VaIN2/3, AIN2/3 and PIN 2/3, respectively. The most common treatment for VIN2/3 (53.5%), VaIN2/3 (47.4%) and AIN2/3 (94.4%) was surgical excision, whereas PIN2/3 patients were more commonly treated by laser excision (44.3%).

The estimated cost per patient for managing VIN2/3, VaIN2/3, AIN2/3 and PIN2/3 based on the use of resources reported by surveyed specialists was 3924€, 3554€, 3985€ and 2722€, respectively.

### Estimation of the burden of diseases associated to the 5 additional types of the 9vHPV in Spain

The number of cases potentially attributable to the 5 additional types and associated costs have been estimated through the information presented in Tables 2 and 3 and costs of managing VIN2/3, VaIN2/3, AIN2/3 and PIN2/3 estimated through surveys to specialists, and are reported in Table 4.

**Table 4:** Number of annual cases of each lesion attributable to HPV genotypes targeted by the 4-valent and 9-valent vaccines and associated costs

Lesion	Cases attributable to HPV 6/11/16/18 and associated costs		Cases attributable to HPV 6/11/16/18/31/33/45/52/58 and associated costs		Cases attributable to HPV 31/33/45/52/58 and associated costs (a)	
	Number of annual cases	Costs (€ 2017)	Number of annual cases	Costs (€ 2017)	Number of annual cases	Costs (€ 2017)
Genital warts (b)	49,251	51.04 Mill €	49,251	51.04 Mill €	0	0.00 Mill €
CIN 2/3	15,285	35.50 Mill €	27,648	64.22 Mill €	12,363	28.72 Mill €
VIN2/3	1001	3.93 Mill €	1162	4.56 Mill €	161	0.63 Mill €
VaIN 2/3	155	0.55 Mill €	186	0.66 Mill €	31	0.11 Mill €
AIN 2/3 (b)	149	0.59 Mill €	162	0.65 Mill €	13	0.05 Mill €
PIN 2/3	218	0.59 Mill €	247	0.67 Mill €	29	0.08 Mill €
Cervical cancer (c)	1730	15.15 Mill €	2110	18.48 Mill €	380	3.33 Mill €
Vulvar cancer (d)	94	1.22 Mill €	110	1.43 Mill €	16	0.21 Mill €
Vaginal cancer (c)	50	0.54 Mill €	66	0.71 Mill €	16	0.17 Mill €
Anal cancer (b)(c)(e)	264	2.02 Mill €	289	2.21 Mill €	25	0.19 Mill €
Penile cancer (c)(e)	98	0.70 Mill €	111	0.79 Mill €	13	0.09 Mill €
Oral cavity (b)(c)(e)	131	1.03 Mill €	147	1.15 Mill €	16	0.12 Mill €
Nasopharynx (b)(c)(e)	27	0.21 Mill €	32	0.25 Mill €	5	0.04 Mill €
Oropharynx cancer (b)(c)(e)	258	1.94 Mill €	272	2.05 Mill €	14	0.10 Mill €
Hypopharynx (b)(c)(e)	28	0.23 Mill €	34	0.28 Mill €	6	0.05 Mill €
Pharynx cancer (b)(c)(e)	39	0.30 Mill €	39	0.30 Mill €	0	0.00 Mill €
Larynx cancer (b)(c)(e)	132	1.15 Mill €	171	1.49 Mill €	39	0.34 Mill €
TOTAL	68,910	116.7 Mill €	82,038	150.9 Mill €	13,128	34.24 Mill €

(a) Difference between the number of cases of each lesion attributable to the genotypes targeted by the 9-valent and 4-valent vaccines and associated costs

(b) Includes both men and women

(c) Genotypes 6 and 11 not included

(d) HPV attributable fraction estimated on HPV DNA and p16 positivity

(e) Only hospitalization costs were considered

*AIN* anal intraepithelial neoplasia; *CIN* cervical intraepithelial neoplasia; *HPV* human papillomavirus; mill.: million; *PIN* penile intraepithelial neoplasia; *VaIN* vaginal intraepithelial neoplasia; *VIN* vulvar intraepithelial neoplasia

Overall, new annual cases of diseases attributable to types targeted by the 4vHPV and 9vHPV represent more than 116 and 150 million € for the Spanish NHS, respectively.

The two type of lesions that represent the greatest burden are genital warts (49,251 cases due to genotypes included in both vaccines, with 51.0 million € related costs) and CIN2/3 (15,285 and 27,648 new cases potentially preventable by the 4vHPV and 9vHPV, representing 35.5 and 64.2 million €, respectively). Within malignancies, cervical cancer is the tumor with the highest burden (1730 and 2110 new cases related to oncogenic genotypes from the 4vHPV and 9vHPV, representing 15.15 and 18.48 million €, respectively) (Table 4).

## Discussion

To the best of our knowledge, the present study estimates, for the first time, the epidemiological and economic burden of HPV-related diseases in Spain, as well as the number of cases associated to types targeted by the 4vHPV and 9vHPV and those associated to the 5 additional types from the new vaccine, considering all diseases for which robust studies have proved HPV implication both in men and women [2, 3]. Moreover, this is also the first time that direct cost of managing VIN2/3, VaIN2/3, AIN2/3 and PIN 2/3 for the Spanish NHS has been estimated. Studies recently published by Hartwig et al. estimated the epidemiological burden and potential benefit of a 9vHPV associated to genital warts and precancerous lesions and cancer of cervix, vulva, vagina and anus in men and women in Europe. This study also extrapolated the European data to 31 European countries including Spain. The number of new annual cases and those attributable to HPV infection, and to genotypes targeted by 4vHPV and 9vHPV for those lesions included in the European study, are comparable to those reported herein [2, 3].

One of the strengths of the present study is that the number of new annual cases for each cancer type was estimated based on data extracted from the Cancer Spanish Registry REDECAN [12], which meets the International Agency for Research in Cancer quality standard and contains data from 15 registries geographically distributed across all Spanish territory, therefore providing accuracy to our estimation.

In addition, the fraction of each lesion attributable to HPV and to the specific genotypes has been calculated applying data from robust studies that use three different markers for viral presence: viral DNA, mRNA, and overexpression of p16, showing high levels of correlation between the different markers [2, 7–10, 21–23], and which in a previous literature review, proved to be consistent with that reported for Spanish population [6] (Table 1).

Furthermore, the economic burden of HPV-related diseases estimated herein, was based on the available literature regarding the direct cost of each type of lesion, except for precancerous lesions other than cervix, for which a specific survey was designed to investigate the management of VIN2/3, VaIN2/3, AIN 2/3 and PIN 2/3, which was then transformed into costs using public available prices for each intervention. These surveys have allowed estimating the cost of managing this type of lesion for the Spanish NHS for the first time.

Another strength of the present study is that, both the methodology and results have been validated by a panel of experts, including the references and methodology used to estimate the number of cases of each disease attributable to HPV and types targeted by both vaccines, the survey used to estimate the use of resources of VIN2/3, VaIN2/3, AIN 2/3 and PIN 2/3 management and costs applied and the final epidemiologic and economic burden estimated.

However, the present study also entails some limitations derived from the studies estimating costs associated to each disease identified through the literature review [6], for instance, the methodology applied and resources considered differ across studies, which lead to the estimated costs not being completely comparable. For example, for those studies estimating the cost of anal, penile and head and neck cancers, only hospitalization costs were considered [29, 30]. Hospitalizations are usually associated to the most severe forms of the diseases, mainly those that require invasive surgical treatment, and may not represent the total burden of the disease. Also, it is worth mentioning that the only reference available estimating the cost of vulvar and vaginal cancer in Spain was a conference abstract for which no full manuscript was identified [28]. Any of the studies that estimate costs differentiates the economic burden of the diseases according to disease stage.

Additionally, although the most robust European data regarding HPV attribution fraction has been used in the present study, the lack of specific and robust Spanish data of HPV attribution in some locations represents a limitation. As aforementioned, a previous literature review based on Spanish studies, proved that the attributable fraction of HPV and types targeted by 4vHPV and 9vHPV vaccines in Spain was consistent with that reported by International studies for CIN2/3, cervical cancer, vulvar cancer and vaginal cancer. However, we assume that for the rest of locations where evidence was not available, international data could be also extrapolated, although we cannot exclude slight differences between HPV circulations among different countries [6].

Moreover, this study only considers high grade lesions and carcinomas in the different locations. However, other glandular lesions, such as adenocarcinoma in situ (AIS) are also related to HPV. These type of lesions are difficult to prevent through cervical screening, due to their location, but HPV vaccines have shown to be effective in its prevention; i.e. in the per protocol population, 9vVPH efficacy to prevent CIN2/3, adenocarcinoma in situ, and cervical cancer due to the 5 additional types was 96.3% (CI 79.5–99.8) [4].

Furthermore, all costs have been transformed to 2017 € according to the CPI evolution, so that they were comparable across studies. It is worth noting these costs include pharmacological treatments whose prices don’t necessarily may have evolved in the same manner as the CPI. Nevertheless, this transformation should not entail a major bias in the cost estimation, as pharmacological treatments are expected to represent a small percentage of the total cost of managing these type of patients in comparison with the costs associated to hospitalizations, visits to specialists and surgical or laser interventions.

In addition, surveys used to estimate the cost for managing VIN2/3, VaIN2/3, AIN 2/3 and PIN 2/3 also entail some limitations, for instance, the fact that costs are estimated based on the answers of a limited group of experts that were not chosen in order to ensure geographic representativeness, as well as the fact that data was collected aggregately, could constitute a bias.

In spite of the aforementioned limitations, the present study highlights the significant epidemiologic and economic burden associated to HPV-related diseases for the Spanish National Health Service. In 2016, 49,251, 29,405 and 3381 cases of new genital warts, precancerous lesions and cancers, respectively, were estimated to be attributable to types targeted by the 9vHPV. Among which, 12,597 (42,8%) and 530 (15,7%), precancerous lesions and cancers could be potentially associated to the 5 additional types from the 9vHPV.

In addition, a total cost of 150.9 million € has been estimated for managing new annual cases of diseases associated to the types targeted by the 9vHPV (34.24 million € more than those associated to the 4vHPV). Except for genital warts and pharynx, the 5 additional types included in the 9vHPV are responsible for a significant fraction of HPV-related diseases in Spain. Particularly, CIN2/3 and cervical cancer are the type of lesions for which a greater contribution of these 5 types and the consequent costs associated may be expected.

The epidemiologic burden reported herein is expected to be reduced in the future, as a consequence of the current national vaccination program against HPV. In 2016, vaccination coverage rate achieved through this program was estimated at 77.8% for 12-year-old girls [31]. In addition, over the last years, some of the regional vaccination calendars have included vaccination against HPV for special high risk groups, i.e. women under conization, or immunocompromised for which no coverage data has been reported yet [32]. However, when considering vaccination of the adult female population out of the national vaccination program, it is estimated to be far below 10%, much lower than that reported in other countries where systematic catch up of adult women have been put in place [33].

## Conclusion

Our results clearly show the great burden of diseases and costs related to the types included in the 9vHPV. Overall, the total annual cost of these diseases is estimated at 150.9 million €, and affect not only females, but also males, which reinforces the need for considering the primary prevention of HPV related diseases in both genders.

## Additional file

Additional file 1: **Table S1.** Full list of search strategies executed on the 25^th^ of June of 2017 in Medline, Embase and Cochrane through the OVID platform: Medline Search strategy. **Table S2.** Use of resource survey for specialists. **Table S3.** Studies estimating direct costs of genital warts, precancerous lesions and cancer of cervical and vaginal, vulvar, anal, penile and head and neck cancers in Spain. **Table S4.** Costs of the interventions and resources, assumptions and sources applied to transform the use of resources from surveys to specialists to cost per patient with VIN2/3, VaIN2/3, AIN2/3 and PIN2/3. (PDF 682 kb)

## Abbreviations

4vHPV: 4-valent human papillomavirus vaccine
5-FU: 5-fluorouracil
9vVPH: 9-valent human papillomavirus vaccine
AIN: Anal intraepithelial neoplasia
CIN: Cervical intraepithelial neoplasia
CPI: Consumer price index
DNA: Deoxyribonucleic acid
GP: General practitioner
HPV: Human papillomavirus
ICD: International Classification of Diseases
IR: Infrared
LEEP: Loop electrosurgical excision procedure
LLETZ: Large loop excision on the transformation zone
Mill.: Million
NHS: National Health Service
NMR: Nuclear magnetic resonance
PIN: Penile intraepithelial neoplasia
VaIN: Vaginal intraepithelial neoplasia
VIN: Vulvar intraepithelial neoplasia
