# Supplementary material for: Epidemiologic and economic burden of HPV diseases in Spain: implication of additional 5 types from the 9-valent vaccine
Source: Infect Agent Cancer. 2018 May 2;13:15. doi: 10.1186/s13027-018-0187-4 (PMC5930836; doi:10.1186/s13027-018-0187-4)
Supplement: Supplementary file 1 — Table S1. Full list of search strategies executed on the 25th of June of 2017 in Medline, Embase and Cochrane through the OVID platform: Medline Search strategy. Table S2. Use of resource survey for specialists. Table S3. Studies estimating direct costs of genital warts, precancerous lesions and cancer of cervical and vaginal, vulvar, anal, penile and head and neck cancers in Spain. Table S4. Costs of the interventions and resources, assumptions and sources applied to transform the use of resources from surveys to specialists to cost per patient with VIN2/3, VaIN2/3, AIN2/3 and PIN2/3. (PDF 682 kb) [file 13027_2018_187_MOESM1_ESM.pdf]

Adittional electronic material

Table S1. Full list of search strategies executed on the 25th of June of 2017 in Medline, Embase and Cochrane through the OVID platform:

Medline Search strategy

#	Search term
1	exp Cancer of Cervix/
2	((cervi$ or uter$) adj3 (cancer$ or neoplasm$ or neoplasia or tumour$ or tumor$ or carcinoma or adenocarcinoma or malingnan$ or metasta$)).mp.
3	Or/1-2
4	exp "Head and Neck Neoplasms"/
5	exp Oropharyngeal Neoplasms/
6	((head$ or neck$ or oro$ $pharyn$) adj3 (cancer$ or neoplasm$ or neoplasia or tumour$ or tumor$ or carcinoma or adenocarcinoma or malingnan$ or metasta$)).mp.
7	or/4-6
8	((precancer$ or pre-cancer$ or neoplas$ or dysplasia or lesion$ or premalignan$ or malignan$ or cancer$ or carcinoma$ or intraepithelial) adj3 (cervi$ or vulva$ or vagina$ or peni$ or anal or anus)).mp.
9	((precancer$ or pre-cancer$ or dysplasia or premalignan$ or intraepithelial) adj3 lesion$).mp.
10	(cin or cin2* or cin3* or cin1* or VAIN or VAIN1* or VAIN2* or VAIN3* or VIN or VIN1* or VIN2* or VIN3* or PeIN or PeIN1* or PeIN2* or PeIN3* or AIN or AIN1* or AIN2* or AIN3*).tw.
11	exp Precancerous Conditions/
12	exp cervical intraepithelial neoplasia/
13	exp uterine cervical dysplasia/
14	vaginal intraepithelial neoplasia {Including Related Terms}
15	penile intraepithelial neoplasia {Including Related Terms}
16	anal intraepithelial neoplasia {Including Related Terms}
17	vulvar intraepithelial neoplasia {Including Related Terms}
18	Or/8-17
19	exp Vaginal Neoplasms/
20	(vagin$ adj3 (cancer$ or neoplasm$ or neoplasia or tumour$ or tumor$ or carcinoma or adenocarcinoma or malingnan$ or metasta$)).mp.
21	Or/19-20
22	exp vulva cancer/
23	(vulv$ adj3 (cancer$ or neoplasm$ or neoplasia or tumour$ or tumor$ or carcinoma or adenocarcinoma or malingnan$ or metasta$)).mp.
24	Or/22-23
25	exp Anus Neoplasms/
26	((anus or anal) adj3 (cancer$ or neoplasm$ or neoplasia or tumour$ or tumor$ or carcinoma or adenocarcinoma or malingnan$ or metasta$)).mp.
27	or/25-26
28	exp Penile Neoplasms/
29	(peni$ adj3 (cancer$ or neoplasm$ or neoplasia or tumour$ or tumor$ or carcinoma or adenocarcinoma or malingnan$ or metasta$)).mp.
30	or/28-29

31	exp Verruca/
32	exp Condylomata Acuminata/
33	(($genit$ or ana$ or ano$ or vener$) adj3 wart$).mp.
34	or/31-33
35	Economics/
36	"costs and cost analysis"/
37	Cost allocation/
38	Cost-benefit analysis/
39	Cost control/
40	Cost savings/
41	Cost of illness/
42	Cost sharing/
43	"deductibles and coinsurance"/
44	Medical savings accounts/
45	Direct service cost/
46	Health care costs/
47	Drug costs/
48	Employer health costs/
49	Hospital costs/
50	Health expenditures/
51	Capital expenditures/
52	Value of life/
53	exp economics, hospital/
54	cost effectiveness analys$.mp.
55	exp economics, medical/
56	Economics, nursing/
57	Economics, pharmaceutical/
58	exp "fees and charges"/
59	exp budgets/
60	(low adj cost).mp.
61	(economic$ and (evaluat$ or analys$ or model$)).mp.
62	(high adj cost).mp.
63	(health?care adj cost$).mp.
64	(fiscal or funding or financial or finance).tw.
65	cost benefit analys$.mp.
66	(cost adj estimate$).mp.

67	(cost adj variable).mp.
68	economics, pharmaceutical/
69	(unit adj cost$).mp.
70	(pharmaco adj economic$).mp.
71	health economic$.mp.
72	economic aspect$.mp.
73	(cba or cea or cua).mp.
74	(econom$ or cost$ or pric$ or pharmacoeconomic$).mp.
75	cost minimi?ation analys$.mp.
76	(cost$ or (cost$ adj2 (effective$ or utili$ or benefit$ or minimi$ or stud$ or effic$ or effect$))).mp.
77	cost utili$ analys$.mp.
78	cost utili$ analys$.mp.
79	cost of illness.mp.
80	economics, nursing/
81	exp economics, medical/
82	exp economics, hospital/
83	(economic$ or pharmacoeconomic$ or price$ or pricing).tw.
84	Or/35-83
85	(Spanish or spain or espagne or espana or spagna or spanien or iberia$).in,hw,mp.
86	(catalunya or catalonia or catalogne or cataluna or catala or barcelon$ or tarragona or lleida or lerida or girona or gerona or sabadell or hospitalet or $hospitalet or (Valencia$ or castello$ or alacant or alicant$)).in,hw,mp.
87	(Murcia or (cartagen$ not indias) or (andalu$ or sevill$ or granad$ or huelva or almeria or cadiz or jaen or malaga or (cordoba not argentin$)) or (extremadura or caceres or badajoz or madrid) or (castilla or salamanca or zamora or valladolid or segovia or soria or palencia or avila or burgos) or (leon not (france or clermont or rennes or lyon or USA or mexic$))).in,hw,mp.
88	(galicia or gallego or compostela or vigo or corun$ or ferrol or orense or ourense or pontevedra or lugo or (oviedo or gijon or asturia$) or (cantabr$ or santander) or (vasco or euskadi or basque or bilbao or bilbo or donosti$ or san sebastian or vizcaya or bizkaia or guipuzcoa or gipuzkoa or alava or araba or vitoria or gasteiz) or (navarr$ or nafarroa or pamplona or iruna or irunea) or (logron$ or rioj$) or (aragon$ or zaragoza or teruel or huesca)).in,hw,mp.
89	(mancha or ciudad real or albacete or cuenca or (toledo not (ohio or us or usa or OH)) or (guadalajara not mexic$) or (balear$ or mallorca or menorca or Ibiza or eivissa) or (palmas or lanzarote or canari$ or tenerif$) or (ceuta or melilla) or ((osasunbide$ or osakidetza or insalud or sergas or catsalut or sespa or osasunbidea or imsalud or sescam or ib) adj1 salud)).in,hw,mp.
90	Or/85-89
91	3 and 84 and 90
92	7 and 84 and 90
93	18 and 84 and 90
94	21 and 84 and 90
95	24 and 84 and 90
96	27 and 84 and 90
97	30 and 84 and 90
98	34 and 84 and 90
99	Or/91-98

**100**	**limit 99 to (yr="1995 -Current" and (english or spanish))**

Embase Search strategy

#	Search term
1	exp cervical cancer/
2	exp uterine cervical neoplasms/
3	exp Cervix Neoplasms/
4	exp Uterine Cervical Cancer/
5	((cervi$ or uter$) adj3 (cancer$ or neoplasm$ or neoplasia or tumour$ or tumor$ or carcinoma or adenocarcinoma or malingnan$ or metasta$)).mp.
6	or/1-5
7	exp "Head and Neck Neoplasms"/
8	exp "Head and Neck Cancer"/
9	exp Head Cancer/
10	exp Neck Cancer/
11	exp Oropharyngeal Neoplasms/
12	exp Oropharyngeal Cancer/
13	exp Oropharynx Cancer/
14	((head$ or neck$ or oro$ $pharyn$) adj3 (cancer$ or neoplasm$ or neoplasia or tumour$ or tumor$ or carcinoma or adenocarcinoma or malingnan$ or metasta$)).mp.
15	or/7-14
16	((precancer$ or pre-cancer$ or neoplas$ or dysplasia or lesion$ or premalignan$ or malignan$ or cancer$ or carcinoma$) adj3 (cervi$ or vulva$ or vagina$ or peni$ or anal or anus)).mp.
17	((precancer$ or pre-cancer$ or dysplasia or premalignan$) adj3 lesion$).mp.
18	(cin or cin2* or cin3* or cin1* or VAIN or VAIN1* or VAIN2* or VAIN3* or VIN or VIN1* or VIN2* or VIN3* or PeIN or PeIN1* or PeIN2* or PeIN3* or AIN or AIN1* or AIN2* or AIN3*).tw.
19	vulvar intraepithelial neoplasia {Including Related Terms}
20	vaginal intraepithelilal neoplasia {Including Related Terms}
21	anal intraepithelial neoplasia {Including Related Terms}
22	penile intraepithelial neoplasia {Including Related Terms}
23	exp cervical intraepithelial neoplasia/
24	exp uterine cervical dysplasia/
25	or/16-24
26	exp Vaginal Neoplasms/
27	exp Vagina Cancer/
28	exp Vaginal cancer/
29	(vagin$ adj3 (cancer$ or neoplasm$ or neoplasia or tumour$ or tumor$ or carcinoma or adenocarcinoma or malingnan$ or metasta$)).mp.

30	or/26-29
31	exp vulva cancer/
32	exp Vulvar Neoplasms/
33	(vulv$ adj3 (cancer$ or neoplasm$ or neoplasia or tumour$ or tumor$ or carcinoma or adenocarcinoma or malingnan$ or metasta$)).mp.
34	or/31-33
35	exp Anus Neoplasms/
36	exp Anal Cancer/
37	((anus or anal) adj3 (cancer$ or neoplasm$ or neoplasia or tumour$ or tumor$ or carcinoma or adenocarcinoma or malingnan$ or metasta$)).mp.
38	or/35-37
39	exp Penile Neoplasms/
40	exp Penile Cancer/
41	exp Penis Cancer/
42	(peni$ adj3 (cancer$ or neoplasm$ or neoplasia or tumour$ or tumor$ or carcinoma or adenocarcinoma or malingnan$ or metasta$)).mp.
43	or/39-42
44	exp Verruca/
45	exp Condylomata Acuminata/
46	exp Genital Warts/
47	exp Venereal Warts/
48	(($genit$ or ana$ or ano$ or vener$) adj3 wart$).mp.
49	or/44-48
50	Economics/
51	"costs and cost analysis"/
52	Cost allocation/
53	Cost-benefit analysis/
54	Cost control/
55	Cost savings/
56	Cost of illness/
57	Cost sharing/
58	"deductibles and coinsurance"/
59	Medical savings accounts/
60	Direct service cost/
61	Health care costs/
62	Drug costs/
63	Employer health costs/
64	Hospital costs/
65	Health expenditures/

66	Capital expenditures/
67	Value of life/
68	exp economics, hospital/
69	cost effectiveness analys$.mp.
70	exp economics, medical/
71	Economics, nursing/
72	Economics, pharmaceutical/
73	exp "fees and charges"/
74	exp budgets/
75	(low adj cost).mp.
76	(economic$ and (evaluat$ or analys$ or model$)).mp.
77	(high adj cost).mp.
78	(health?care adj cost$).mp.
79	(fiscal or funding or financial or finance).tw.
80	cost benefit analys$.mp.
81	(cost adj estimate$).mp.
82	(cost adj variable).mp.
83	economics, pharmaceutical/
84	(unit adj cost$).mp.
85	(pharmaco adj economic$).mp.
86	health economic$.mp.
87	economic aspect$.mp.
88	(cba or cea or cua).mp.
89	(econom$ or cost$ or pric$ or pharmacoeconomic$).mp.
90	cost minimi?ation analys$.mp.
91	(cost$ or (cost$ adj2 (effective$ or utili$ or benefit$ or minimi$ or stud$ or effic$ or effect$))).mp.
92	cost utili$ analys$.mp.
93	cost utili$ analys$.mp.
94	cost of illness.mp.
95	economics, nursing/
96	exp economics, medical/
97	exp economics, hospital/
98	(economic$ or pharmacoeconomic$ or price$ or pricing).tw.
99	or/50-98
100	(Spanish or spain or espagne or espana or spagna or spanien or iberia$).in,hw,mp.
101	(catalunya or catalonia or catalogne or cataluna or catala or barcelon$ or tarragona or lleida or lerida or girona or gerona or sabadell or hospitalet or $hospitalet or (Valencia$ or castello$ or alacant or alicant$)).in,hw,mp.

102	(Murcia or (cartagen$ not indias) or (andalu$ or sevill$ or granad$ or huelva or almeria or cadiz or jaen or malaga or (cordoba not argentin$)) or (extremadura or caceres or badajoz or madrid) or (castilla or salamanca or zamora or valladolid or segovia or soria or palencia or avila or burgos) or (leon not (france or clermont or rennes or lyon or USA or mexic$))).in,hw,mp.
103	(galicia or gallego or compostela or vigo or corun$ or ferrol or orense or ourense or pontevedra or lugo or (oviedo or gijon or asturia$) or (cantabr$ or santander) or (vasco or euskadi or basque or bilbao or bilbo or donosti$ or san sebastian or vizcaya or bizkaia or guipuzcoa or gipuzkoa or alava or araba or vitoria or gasteiz) or (navarr$ or nafarroa or pamplona or iruna or irunea) or (logron$ or rioj$) or (aragon$ or zaragoza or teruel or huesca)).in,hw,mp.
104	(mancha or ciudad real or albacete or cuenca or (toledo not (ohio or us or usa or OH)) or (guadalajara not mexic$) or (balear$ or mallorca or menorca or Ibiza or eivissa) or (palmas or lanzarote or canari$ or tenerif$) or (ceuta or melilla) or ((osasunbide$ or osakidetza or insalud or sergas or catsalut or sespa or osasunbidea or imsalud or sescam or ib) adj1 salud)).in,hw,mp.
105	or/100-104
106	6 and 99 and 105
107	15 and 99 and 105
108	25 and 99 and 105
109	30 and 99 and 105
110	34 and 99 and 105
111	38 and 99 and 105
112	43 and 99 and 105
113	49 and 99 and 105
114	or/106-113
**115**	**limit 114 to ((english or spanish) and yr="1995 -Current")**

Cochrane search strategy

#	Término de busqueda
1	[Cervical Cancer] {Including Limited Related Terms}
2	[Cervical Neoplasms] {Including Limited Related Terms}
3	[Cervix Cancer] {Including Limited Related Terms}
4	[Cervix Neoplasm] {Including Limited Related Terms}
5	[Uterine Cervical Cancer] {Including Limited Related Terms}
6	[cervix neoplasia] {Including Limited Related Terms}
7	((cervi$ or uter$) adj3 (cancer$ or neoplasm$ or neoplasia or tumour$ or tumor$ or carcinoma or adenocarcinoma or malingnan$ or metasta$)).mp.
8	or/1-7
9	[Head and Neck Neoplasms] {Including Limited Related Terms}
10	[Head and Neck Cancer] {Including Limited Related Terms}
11	[Head Cancer] {Including Limited Related Terms}
12	[neck cancer] {Including Limited Related Terms}
13	[Oropharyngeal Neoplasms] {Including Limited Related Terms}
14	[Oropharyngeal Cancer] {Including Limited Related Terms}
15	[Oropharynx Cancer] {Including Limited Related Terms}
16	[oropharynx neoplasia] {Including Limited Related Terms}
17	[head and neck neoplasia] {Including Limited Related Terms}
18	((head$ or neck$ or oro$ $pharyn$) adj3 (cancer$ or neoplasm$ or neoplasia or tumour$ or tumor$ or carcinoma or adenocarcinoma or malingnan$ or metasta$)).mp.
19	or/9-18

20	((precancer$ or pre-cancer$ or neoplas$ or dysplasia or lesion$ or premalignan$ or malignan$ or cancer$ or carcinoma$ or intraepithelial) adj3 (cervi$ or vulva$ or vagina$ or peni$ or anal or anus)).mp.
21	(cin or cin2* or cin3* or cin1* or VAIN or VAIN1* or VAIN2* or VAIN3* or VIN or VIN1* or VIN2* or VIN3* or PeIN or PeIN1* or PeIN2* or PeIN3* or AIN or AIN1* or AIN2* or AIN3*).tw.
22	((precancer$ or pre-cancer$ or dysplasia or premalignan$ or intraepithelial) adj3 lesion$).mp.
23	[precancerous lession] {Including Limited Related Terms}
24	[precancerous lesions] {Including Limited Related Terms}
25	[vulvar intraepithelial neoplasia] {Including Limited Related Terms}
26	[vaginal intraepithelial neoplasia] {Including Limited Related Terms}
27	[anal intraepithelial neoplasia] {Including Limited Related Terms}
28	[penile intraepithelial neoplasia] {Including Limited Related Terms}
29	[cervical intraepithelial neoplasia] {Including Limited Related Terms}
30	[uterine cervical neoplasms] {Including Limited Related Terms}
31	or/20-30
32	[Vaginal Neoplasm] {Including Limited Related Terms}
33	[vagina neoplasm] {Including Limited Related Terms}
34	[vagina neoplasia] {Including Limited Related Terms}
35	[vaginal neoplasia] {Including Limited Related Terms}
36	(vagin$ adj3 (cancer$ or neoplasm$ or neoplasia or tumour$ or tumor$ or carcinoma or adenocarcinoma or malingnan$ or metasta$)).mp.
37	or/32-36
38	[vulva cancer] {Including Limited Related Terms}
39	[Vulvar Neoplasms] {Including Limited Related Terms}
40	[Vulva neoplasia] {Including Limited Related Terms}
41	[vulvar neoplasia] {Including Limited Related Terms}
42	(vulv$ adj3 (cancer$ or neoplasm$ or neoplasia or tumour$ or tumor$ or carcinoma or adenocarcinoma or malingnan$ or metasta$)).mp.
43	or/38-42
44	[Anus Neoplasms] {Including Limited Related Terms}
45	[Anal Cancer] {Including Limited Related Terms}
46	[anus neoplasia] {Including Limited Related Terms}
47	[anal neoplasia] {Including Limited Related Terms}
48	((anus or anal) adj3 (cancer$ or neoplasm$ or neoplasia or tumour$ or tumor$ or carcinoma or adenocarcinoma or malingnan$ or metasta$)).mp.
49	or/44-48
50	[Penile Cancer] {Including Limited Related Terms}
51	[Penis Cancer] {Including Limited Related Terms}
52	[penis neoplasia] {Including Limited Related Terms}
53	[penile neoplasia] {Including Limited Related Terms}
54	(peni$ adj3 (cancer$ or neoplasm$ or neoplasia or tumour$ or tumor$ or carcinoma or adenocarcinoma or malingnan$ or metasta$)).mp.
55	or/50-54
56	[Verruca] {Including Limited Related Terms}
57	[Condylomata Acuminata] {Including Limited Related Terms}

58	[Genital Warts] {Including Limited Related Terms}
59	[Venereal Warts] {Including Limited Related Terms}
60	(($genit$ or ana$ or ano$ or vener$) adj3 wart$).mp.
61	or/56-60
62	[Economics] {Including Limited Related Terms}
63	[pharmacoeconomics] {Including Limited Related Terms}
64	pharmacoeconomic$.mp.
65	health economic$.mp.
66	economic aspect$.mp.
67	economic$.mp.
68	economic evaluation.mp.
69	(economic$ and (evaluat$ or analys$ or model$)).mp.
70	cost utili$ analys$.mp.
71	(cost$ or (cost$ adj2 (effective$ or utili$ or benefit$ or minimi$ or stud$ or effic$ or effect$))).mp.
72	cost of illness.mp.
73	cost minimi?ation analys$.mp.
74	cost effectiveness analys$.mp.
75	cost benefit analys$.mp.
76	(cba or cea or cua).mp.
77	(cost$ adj3 estimate$).mp.
78	(unit adj3 cost$).mp.
79	budget$.mp.
80	expenditure$.mp.
81	(decision adj2 (tree$ or analys$ or model$)).mp.
82	markov$.mp.
83	Or/62-82
84	(Spanish or spain or espagne or espana or spagna or spanien or iberia$).mp.
85	(catalunya or catalonia or catalogne or cataluna or catala or barcelon$ or tarragona or lleida or lerida or girona or gerona or sabadell or hospitalet or $hospitalet or (Valencia$ or castello$ or alacant or alicant$)).mp.
86	(Murcia or (cartagen$ not indias) or (andalu$ or sevill$ or granad$ or huelva or almeria or cadiz or jaen or malaga or (cordoba not argentin$)) or (extremadura or caceres or badajoz or madrid) or (castilla or salamanca or zamora or valladolid or segovia or soria or palencia or avila or burgos) or (leon not (france or clermont or rennes or lyon or USA or mexic$))).mp.
87	(galicia or gallego or compostela or vigo or corun$ or ferrol or orense or ourense or pontevedra or lugo or (oviedo or gijon or asturia$) or (cantabr$ or santander) or (vasco or euskadi or basque or bilbao or bilbo or donosti$ or san sebastian or vizcaya or bizkaia or guipuzcoa or gipuzkoa or alava or araba or vitoria or gasteiz) or (navarr$ or nafarroa or pamplona or iruna or irunea) or (logron$ or rioj$) or (aragon$ or zaragoza or teruel or huesca)).mp.
88	(mancha or ciudad real or albacete or cuenca or (toledo not (ohio or us or usa or OH)) or (guadalajara not mexic$) or (balear$ or mallorca or menorca or Ibiza or eivissa) or (palmas or lanzarote or canari$ or tenerif$) or (ceuta or melilla) or ((osasunbide$ or osakidetza or insalud or sergas or catsalut or sespa or osasunbidea or imsalud or sescam or ib) adj1 salud)).mp.
89	Or/84-88
90	8 or 19 or 31 or 37 or 43 or 49 or 55 or 61
**91**	**83 and 89 and 90**

Table S2. Use of resource survey for specialists

Section 1: Screener questions

S1. Please, select your medical specialty:

a. Gynecology	
b. Urology	
c. Coloproctology	
d. Surgery	
e. Internal Medicine	
f. Other specialty:	

S2. Please, mark whether you have visited at least the number of patients indicated for each pathology included in the table below during the last 18 months if you can provide information about the treatment used. In case you have managed more than one pathology, choose 2 of them as maximum (those for which you have visited the highest number of patients)

**Pathologies**	
a. 5 o more cases of vaginal intraepthelial neoplasia grade 2 or 3 (high grade)	
b. 3 o more cases of vulvar intraepthelial neoplasia grade 2 or 3 (high grade)	
c. 3 o more cases of anal intraepthelial neoplasia grade 2 or 3 (high grade)	
d. 5 o more cases of penile intraepthelial neoplasia grade 2 or 3 (high grade in situ carcinoma, Bowen disease, Queyrat erythroplasia or Bowenoide papullosis	
e. I have not visited the minimum required number of patients of any of these diseases during the last 18 month	

Section 2: Number of patients

Q1 Resources used for diagnosis and treatment

Q1. Regarding the number of visits, hospitalizations, diagnosis tests and treatments that were necessary for the diagnosis and treatment of patients for each pathology, please, indicate how many patients required each type of visit, diagnosis test, treatment and procedure.

Vaginal intraepithelial neoplasia, last 5 patients

Visits / hospitalizations / treatments / procedures / diagnosis test	Number of times that the intervention or visit was done	Number of patients
Primary care visit	1	
	2	
	3	
	4 or more	
Specialist visit	1	
	2	
	3	
	4 or more	
Hospitalization days	1	
	2	
	3	
	4 or more	
Emergency visits	1	
	2	
	3	
	4 or more	
Cytologies done	1	
	2	
	3	
	4 or more	
Test HPV	1	
	2	
	3	
	4 or more	
Vaginoscopy	1	
	2	
	3	
	4 or more	
Biopsy	1	
	2	
	3	
	4 or more	
Laser excision, ablation or vaporization	1	
	2	
	3	
	4 or more	
Excision, ablation or surgical exeresis or vaginal colpectomy	1	
	2	
	3	
	4 or more	
Inguinal lymphadenectomy	1	
	2	
	3	
	4 or more	
Total or radical colpectomy	1	
Intracavitary radiation (brachytherapy)	1	
Others, please, specify the treatment or diagnosis test and the number of patients who received it		

Vulvar intraepithelial neoplasia, last 3 patients

Visits / hospitalizations / treatments / procedures / diagnosis test	Number of times that the intervention or visit was done	Number of patients
Primary care visit	1	
	2	
	3	
	4 or more	
Specialist visit	1	
	2	
	3	
	4 or more	
Hospitalization days	1	
	2	
	3	
	4 or more	
Emergency visits	1	
	2	
	3	
	4 or more	
Cytologies done	1	
	2	
	3	
	4 or more	
Test HPV	1	
	2	
	3	
	4 or more	
Vaginoscopy	1	
	2	
	3	
	4 or more	
Biopsy	1	
	2	
	3	
	4 or more	
Laser excision, ablation or vaporization	1	
	2	
	3	
	4 or more	
Excision, ablation or surgical exeresis or partial vulvectomy	1	
	2	
	3	
	4 or more	
Inguinal lymphadenectomy	1	
	2	
	3	
	4 or more	
Total or radical vulvectomy	1	
Topic Imiquimod	1	
Others, please, specify the treatment or diagnosis test and the number of patients who received it		

Anal intraepithelial neoplasia, last 3 patients

Visits / hospitalizations / treatments / procedures / diagnosis test	Number of times that the intervention or visit was done	Number of patients
Primary care visit	1	
	2	
	3	
	4 or more	
Specialist visit	1	
	2	
	3	
	4 or more	
Hospitalization days	1	
	2	
	3	
	4 or more	
Emergency visits	1	
	2	
	3	
	4 or more	
Cytologies done	1	
	2	
	3	
	4 or more	
Test HPV	1	
	2	
	3	
	4 or more	
Vaginoscopy	1	
	2	
	3	
	4 or more	
Biopsy	1	
	2	
	3	
	4 or more	
Cryotherapy	1	
	2	
	3	
	4 or more	
Trichloroacetic acid	1	
	2	
	3	
	4 or more	
Laser excision, ablation or vaporization	1	
	2	
	3	
	4 or more	
Excision, ablation or surgical exeresis or anal resection	1	
	2	
	3	
	4 or more	
Inguinal lymphadenectomy	1	
	2	
	3	
	4 or more	
Colostomy	1	
5% 5-fluorouracilo topic	1	
5% Imiquimod	1	
Others, please, specify the treatment or diagnosis test and the number of patients who received it		

Penile intraepithelial neoplasia, last 3 patients

Visits / hospitalizations / treatments / procedures / diagnosis test	Number of times that the intervention or visit was done	Number of patients
Primary care visit	1	
	2	
	3	
	4 or more	
Specialist visit	1	
	2	
	3	
	4 or more	
Hospitalization days	1	
	2	
	3	
	4 or more	
Emergency visits	1	
	2	
	3	
	4 or more	
Cytologies done	1	
	2	
	3	
	4 or more	
Test HPV	1	
	2	
	3	
	4 or more	
Vaginoscopy	1	
	2	
	3	
	4 or more	
Biopsy	1	
	2	
	3	
	4 or more	
Laser excision, ablation or vaporization	1	
	2	
	3	
	4 or more	
Mohs micrographic surgery	1	
	2	
	3	
	4 or more	
Inguinal lymphadenectomy	1	
	2	
	3	
	4 or more	
5% 5-fluorouracilo topic	1	
5% Imiquimod	1	
Others, please, specify the treatment or diagnosis test and the number of patients who received it		

Table S3. Studies estimating direct costs of genital warts, precancerous lesions and cancer of cervical and vaginal, vulvar, anal, penile and head and neck cancers in Spain.

Reference	Disease	Methodology	Direct costs for the NHS
Castellsagué, 2009 [1]	Genital warts	Retrospective study of 300 patients aged 14-64 from 6 AACC with genital warts related to HPV.	Mean cost per patient: 833€. Total annual cost: 47 million €.
Castellsagué, 2009 [2]	Cervical precancerous lesions (CIN 1/2/3)	Retrospective study with 362,052 women and 65 gynecologists from 6 AACC.	Mean cost per patient of exploration, diagnosis and treatment: CIN 1: 1,115€ // CIN 2: 1,626€ // CIN 3: 2,090€. Total annual cost: 147 million €.
Rash, 2008 [3]	Cervical precancerous lesions (CIN 1/2/3)	Retrospective chart review of women attending their gynecologist with abnormal cytology or colposcopy between June 2005 and May in 5 countries, including Spain (159 with CIN 1, 118 with CIN 2 and 282 with CIN 3).	Mean cost of cytology and histological analysis: CIN1: 1,090.4€ // CIN 2: 1,570.9€ // CIN 3: 1,640.6€
Bayas, 2008 [4]	Cervical carcinoma in situ (CIN 3) and cancer	Retrospective study of hospitalized women due to cervical cancer or CIN 3 in Catalonia during 1999-2002, using data from CMBD and a chart review from Hospital Clínic de Barcelona (172 with cervical cancer and 62 with CIN 3).	Mean cost of hospital treatment per patient: CIN 3: 2,157€ // Cancer: 5,722€. Total annual cost for the Catalan NHS: CIN 3: 990,063€ // Cancer: 3,850,906€
Blade, 2010 [5]	Cervical precancerous lesions (CIN 1/2/3) and cancer.	Multicenter, retrospective study (8 centers) with 849 women with abnormal cervical cytology results (159 with CIN 1, 120 with CIN 2, 295 with CIN 3 and 79 with cervical cancer).	Mean direct costs per patient for diagnosis and treatment: CIN 1: 790.1€ // CIN 2: 1.131.2€ // CIN 3: 1,181.3€ // Cancer: 7,041.7€.
Gil, 2007 [6]	Cervical carcinoma in situ (CIN 3) and cancer.	Retrospective study of CMBD hospital discharge data of cervical cancer (16,604 discharges) and CIN 3 (11,044 discharges). Costs were estimated from corresponding DRG.	Mean direct cost per hospitalized patient: CIN 3: 2,192€ // cervical cancer: 3,098€. Mean total annual cost: CIN 3: 6,042,357€ // Cancer: 12,884,359€
De Juanes, 2010 [7]	Cervical cancer	Retrospective study of hospitalized patients with malignant cervical neoplasm in Madrid AACC between 1999 and 2002 from CMBD data. Use of resources and associated costs were estimated through a chart review of 99 patients' medical records from Hospital 12 de Octubre.	Mean cost per patient of hospital treatment: 5,247€. Total annual cost for Madrid AACC: 3.499.638€
García-Garrido, 2014 [8]	Cervical cancer	Descriptive study to analyze cervical cancer screening in Cantabria between 2006 and 2011 from CMBD data and health areas II, III and IV from Cantabria and data from the oncological radiotherapy department from University Hospital Marqués de Valdecilla.	Total cost of hospitalization radiotherapy and diagnosis for the period 2008-2010: 567,567€.
Rana, 2011 [9] (a,b)	Cervical, vulvar and vaginal cancer	Literature review: EMBASE, PUBMED, Cochrane, Globocan, WHO, SEER and grey literature to explore epidemiology and costs associated to genital cancer related with HPV in women.	Total annual cost of hospitalization of cervical cancer and CIN3: 19 million € (22)
Cortes, 2012 [10] (b)	Vulvar and vaginal cancer	Literature review and interviews to explore about epidemiology and direct and indirect costs of vulvar and vaginal cancer	Direct cost of diagnosis, treatment and hospitalization per patient for: vulvar cancer: 12.470€ // vaginal cancer: 10.235€. Total annual cost: 8.493.058€
Gil-Prieto, 2012 [11]	Anal and penile cancer	Retrospective study from CMBD data of anal and penile cancer hospital discharges between 1997 and 2008. Costs were estimated from the corresponding DRG.	Direct cost per hospitalized patient: anal cancer: 6,968€/men and 6,692€/women // penile cancer: 6,382€ Total annual cost: 4.1 million € in men and 2.8 million € in women // penile cancer 4.1 million de €.
Gil-Prieto, 2012 [12]	Head and neck cancer	Retrospective study from CMBD data of head and neck cancer hospital discharges between 1997 and 2008. Costs were estimated from the	Direct cost per hospitalized patient: oral cavity: 7,141€/men and 7.333€/women // Pharynx: 6.886 €/men and 7.936 €/women // Larynx:

	corresponding DRG.	7.739 €/men and 8.280 €/women // Tonsil: 6.510€/men and 6.393€/women // Tongue: 7.070 €/men and 7.661€/women // Other: 6.595 €/men and 6.675€/women. Total annual cost: 149 million €.

(a) The literature review included cervical, vulvar and vaginal cancer, but only direct costs for cervical cancer were reported.; (b) Conference abstract; AACC: Autonomous community; CIN: Cervical Intraepithelial Neoplasia; CMBD: Basic minimal data set of hospital discharges retrieved and published by the Spanish Ministry of health; DRG: Diagnosis-related groups; HVP: Human Papillomavirus; NHS: National Health Service.

1. Castellsague X, Cohet, C, Puig-Tintore, L M, et al. Epidemiology and cost of treatment of genital warts in Spain. *Eur J Public Health*2009;19:106-10.
2. Castellsague X, Remy V, Puig-Tintore L M, de la Cuesta R S, Gonzalez-Rojas N, Cohet C. Epidemiology and costs of screening and management of precancerous lesions of the cervix in Spain. *J Low Genit Tract Dis* 2009;13:38-45.
3. Rash B, Martin-Hirsch P, Schneider A, et al. Resource use and cost analysis of managing abnormal Pap smears: a retrospective study in five countries. *Eur J Gynaecol Oncol* 2008;29:225-32.
4. Bayas JM, Gil R, San-Martin M, et al. Hospitalizations for cervical cancer and carcinoma in situ in Catalonia during 1999-2002. *Vacunas* 2008;9:144-50.
5. Blade A, Cararach M, Castro M, Catala-Lopez, F, Perez-Escolano I, de Sanjose S. Clinical management of abnormal cytology test results and costs associated with the prevention of cervical cancer in Spain. *J Low Genit Tract Dis* 2010;14:311-8.
6. Gil A, San-Martin M, Gil R, Hernandez V, Ribes J, Gonzalez A. Burden of hospital admissions for cervical cancer in Spain during 1999-2002. *Hum Vaccin*2007;3:276-80.
7. De Juanes JR, Arrazola P, Garcia de Codes A, et al. Hospital admissions for cervical cancer in the autonomous Region of Madrid, 1999-2002. *Progresos de Obstetricia y Ginecologia* 2010;53:297-302.
8. Garcia-Garrido AB, Vazquez-Rodriguez J A, Grande-Gonzalez E, Ramos-Barron M A Coverage and costs of opportunistic screening for cervical cancer in Cantabria (Spain). *Gac Sanit* 2014;28:14-9.
9. Rana C, Mann K, Wadhwa A, Pathak P. Incidence rate and burden of illness associated with human papillomavirus related genital cancers in Spanish women. *Value in Health* 2011;14:A442.
10. Cortes J, Hurtado P, Castellsague X. Burden of disease due to vulvar and vaginal cancers in Spain. *Int J Gynaecol Obstet* 2012;119:S644-S5.
11. Gil-Prieto R, Ester P V, Alvaro-Meca A, Rodriguez M S, De Miguel A G. The burden of hospitalizations for anus and penis neoplasm in Spain (1997-2008). *Hum Vaccin Immunother* 2012;8:201-7.
12. Gil-Prieto R, Ester P V, Alvaro-Meca A, San Martin Rodriguez M, De Miguel A G. The burden of hospitalizations for head and neck neoplasm in Spain (1997-2008): An epidemiologic study. *Human Vaccines and Immunotherapeutics* 2012;8:788-98.

Table S4. Costs of the interventions and resources, assumptions and sources applied to transform the use of resources from surveys to specialists to cost per patient with VIN2/3, VaIN2/3, AIN2/3 and PIN2/3

Intervention/resource	Unitary cost (€ 2017)	Assumptions	Sources
**First GP visit**	66.97 €	For patients with one only visit, this cost has been assumed.	Health Office (2015). Resolution of the 30th of March of 2015, of the Director, that modifies public prices of health services of the Decree 81/2009, of the 16th of June, that establishes public prices for health services offered by the Health Service of the Canary Islands. *Boletín Oficial de Canarias* num. 70, of the 14th of April of 2015.
**Successive GP visits**	28.97 €	For patients with 2 or more visits, the cost of the first visit was assumed for one of them, and the cost corresponding to successive visits was assumed for all the rest.	Health Office (2015). Resolution of the 30th of March of 2015, of the Director, that modifies public prices of health services of the Decree 81/2009, of the 16th of June, that establishes public prices for health services offered by the Health Service of the Canary Islands. *Boletín Oficial de Canarias* num. 70, of the 14th of April of 2015.
**Visit to general and digestive system surgery specialist**	141.56 €	This cost has been assumed for all visits to "coloproctologists"	Health Office (2011) RESOLUTION of the 21st of November of 2011. *Boletín Oficial de Castilla y León*, num. 234, 5th of December of 2011.
**First urology visit**	97.85 €	For patients with one only visit, this cost has been assumed.	National Health Institute (2001). Results of the analytical management of hospitals from INSALUD GECLIF 2000. *Subdirección General de Coordinación Administrativa*, Madrid 2001. (Referred to: INSALUD)
**Successive Urology visits**	58.70 €	For patients with 2 or more visits, the cost of the first visit was assumed for one of them, and the cost corresponding to successive visits was assumed for all the rest.	National Health Institute (2001). Results of the analytical management of hospitals from INSALUD GECLIF 2000. *Subdirección General de Coordinación Administrativa*, Madrid 2001. (Referred to: INSALUD)
**First gynecology visit**	76.12 €	For patients with one only visit, this cost has been assumed.	National Health Institute (2001). Results of the analytical management of hospitals from INSALUD GECLIF 2000. *Subdirección General de Coordinación Administrativa*, Madrid 2001. (Referred to: INSALUD)
**Successive gynecology visit**	45.67 €	For patients with 2 or more visits, the cost of the first visit was assumed for one of them, and the cost corresponding to successive visits was assumed for all the rest.	National Health Institute (2001). Results of the analytical management of hospitals from INSALUD GECLIF 2000. *Subdirección General de Coordinación Administrativa*, Madrid 2001. (Referred to: INSALUD)
**Hospitalization general and digestive system surgery specialist (cost per day)**	825.50 €	For all hospitalizations of AIN2/3, this cost was assumed	Adapted from the Ministry of Health, Social Services and Equality. Health Information Institute (2013). Discharge registry – CMBD 2013. http://pestadistico.inteligenciadegestion.msssi.es/
**Hospitalization urology (cost per day)**	850.55 €	For all hospitalizations of PIN2/3, this cost was assumed	Adapted from the Ministry of Health, Social Services and Equality. Health Information Institute (2013). Discharge registry – CMBD 2013. http://pestadistico.inteligenciadegestion.msssi.es/

**Hospitalization gynecologist (cost per day)**	850.55 €	For all hospitalizations of VIN2/3 and VaIN2/3, this cost was assumed	Adapted from the Ministry of Health, Social Services and Equality. Health Information Institute (2013). Discharge registry – CMBD 2013. http://pestadistico.inteligenciadegestion.msssi.es/
**Emergency visit**	134.01 €		Zumárraga Hospital. (2003). http://www.hospitalzumarraga.org/info/indicadores.htm.
**Cytology**	28.76 €		Osakidetza Public Directorate (2015). Agreement of the 23rd of February of 2015. Book of prices. Available at: http://osakidetza.euskadi.net/r85-ckproc05/es/contenidos/informacion/libro_tarifas/es_libro/tarifas.htm
**DNA HPV test**	86.75 €		Catalan Institute of Health (2013). Resolution SLT/353/2013 of the 13th of February of 2013. *Diari Oficial de la Generalitat de Catalunya* num 6326, 1st of March of 2013.
**Colposcopy**	29.44 €		Catalan Institute of Health (2013). Resolution SLT/353/2013 of the 13th of February of 2013. *Diari Oficial de la Generalitat de Catalunya* num. 6326, 1st of March of 2013.
**Vaginoscopy**	29.44 €	Same cost as “colposcopy” was assumed	Catalan Institute of Health (2013). Resolution SLT/353/2013 of the 13th of February of 2013. *Diari Oficial de la Generalitat de Catalunya* num. 6326, 1st of March of 2013.
**Vulvoscopy**	29.44 €	Same cost as “colposcopy” was assumed	Catalan Institute of Health (2013). Resolution SLT/353/2013 of the 13th of February of 2013. *Diari Oficial de la Generalitat de Catalunya* num. 6326, 1st of March of 2013.
**Anoscopy**	82.88 €		Health Office (2015). Resolution of the 30th of March of 2015, of the Director, that modifies public prices of health services of the Decree 81/2009, of the 16th of June, that establishes public prices for health services offered by the Health Service of the Canary Islands. *Boletín Oficial de Canarias* num. 70, of the 14th of April of 2015.
**Vaginal biopsy**	62.93 €		Catalan Institute of Health (2013). Resolution SLT/353/2013 of the 13th of February of 2013. *Diari Oficial de la Generalitat de Catalunya* num 6326, 1st of March of 2013.
**Vulvar biopsy**	62.93 €		Catalan Institute of Health (2013). Resolution SLT/353/2013 of the 13th of February of 2013. *Diari Oficial de la Generalitat de Catalunya* num. 6326, 1st of March of 2013.
**Anal biopsy**	62.93 €		Catalan Institute of Health (2013). Resolution SLT/353/2013 of the 13th of February of 2013. *Diari Oficial de la Generalitat de Catalunya* num. 6326, 1st of March of 2013.
**Penile biopsy**	122.43 €		Health Office (2015) Order of the 18th of November of 2015. *Boletín Oficial de la Junta de Andalucía* num. 228, 24th of November of 2015.
**Laser excision**	674.66 €	Cost of “tumors fulguration with laser, argon gas and others” was assumed.	Health Office of the Balearic Islands (2014). Resolution of the general director of the health office to modify appendix I of the Order of the Health Office of the 22nd of December of 2006. *Butlletí Oficial de les Illes Balears* num. 89, of the 1st of July of 2014.

**Vaginal surgical excision or partial colpectomy**	1,564.80 €	Cost of "other excision or destruction of vagina" was assumed	Health Office of Navarra- Osasunbidea (2014) Resolution 626/2014 of the 5th of June. *Boletín Oficial de Navarra*, num. 133, 9th of July 2014.
**Vulvar surgical excision or partial vulvectomy**	1,655.42 €	Cost of " other excision or destruction of vulva and perineum" was assumed	Health Office of Navarra- Osasunbidea (2014) Resolution 626/2014 of the 5th of June. *Boletín Oficial de Navarra*, num. 133, 9th of July 2014.
**Anal surgical excision**	1,222.07 €	Cost of "anus excision" was assumed	Health Office of Navarra- Osasunbidea (2014) Resolution 626/2014 of the 5th of June. *Boletín Oficial de Navarra*, num. 133, 9th of July 2014.
**Penile surgical excision**	1,665.45 €	Cost of "major ambulatory surgery, penile procedures" was assumed	Tax and Public Administration Office (2014) Order 17/2014, of the 16th of November of 2014. *Boletín Oficial de la Rioja*, num. 156, 19th of December of 2014.
**Braquitherapy**	987.07 €		Presidency of the Generalitat. (2008). Law 16/2008 of the 22nd of December. *Diari Oficial de la Generalitat Valenciana* num. 5922.
**Cryotherapy**	200.51 €		Catalan Institute of Health. (2008) Order SLT/434/2008 of the 12th of February of 2008. *Diari Oficial de la Generalitat de Catalunya* num. 5076, 22nd of February of 2008.
**NMR**	176.30 €	Cost of "simple study" was assumed	Health Office of the Basque Country (2011). Order of the 14th of December of 2011. Boletín Oficial del País Vasco nº 247, 30 diciembre 2011.
**Conization**	893.20 €		Health Office (2013). Order 731/2013 6th of September of 2013. *Boletín Oficial de la Comunidad de Madrid* num. 215, 10th of September of 2013.
**Cervical culture**	26.70 €		Osakidetza Public Directorate (2015). Agreement of the 23rd of February of 2015. Book of prices. Available at:http://osakidetza.euskadi.net/r85-ckproc05/es/contenidos/informacion/libro_tarifas/es_libro/tarifas.htm
**General analytics**	66.86 €	Cost of "general hematologic and biochemical analytic "was assumed	Health Office of the Balearic Islands (2014). Resolution of the general director of the health office to modify appendix I of the Order of the Health Office of the 22nd of December of 2006. *Butlletí Oficial de les Illes Balears* num. 89, of the 1st of July of 2014.
**Sexually transmitted disease serology**	145.58 €	Cost of "serology" was assumed	Sánchez-Blanco, J.J. y cols. (2004). Economic cost of peripheral blood progenitor cell transplantation in Spain. Med Clin (Barc) 2004;123:401-5. (Referred to: General University Hospital. Morales Meseguer (Murcia))
**Infrared**	5.22 €		Iñigo, V. y cols. (2010). Assistance activity reorientation in a physical medicine and rehabilitation service: Costs analyses. Evidence and cost-effectiveness medicine. Rehabilitación (Madr). 2010;44:145-151.
**Imiquimod**	75.56 €	Cost of Imunocare® (50 mg/g cream 24 bags 250 mg) was assumed	BotPlus
**5-fluorouracil**	12.61	Cost of 1 vial x 100 mL, 50 mg/ml was assumed	BotPlus
**Trichloroacetic acid**	38.13 €	Cost of 100 mL 50% aqueous solution was assumed	Official College of Pharmacists from Barcelona

AIN: anal intraepithelial neoplasia; CMBD: basic minimal data set of hospital discharges retrieved and published by the Spanish Ministry of Health; DNA: deoxyribonucleic acid; GP: general practitioner; HPV: human papillomavirus; NMR: nuclear magnetic resonance; PIN: penile intraepithelial neoplasia; VaIN: vaginal intraepithelial neoplasia; VIN: vulvar intraepithelial neoplasia.
